# Cystathionine γ-lyase and hydrogen sulfide modulates glucose transporter Glut1 expression via NF-κB and PI3k/Akt in macrophages during inflammation

**DOI:** 10.1371/journal.pone.0278910

**Published:** 2022-12-15

**Authors:** Alex Cornwell, Samantha Fedotova, Sara Cowan, Alireza Badiei

**Affiliations:** 1 Department of Biology and Wildlife, College of Natural Science and Mathematics, University of Alaska Fairbanks, Fairbanks, Alaska, United States of America; 2 Department of Veterinary Medicine, College of Natural Science and Mathematics, University of Alaska Fairbanks, Fairbanks, Alaska, United States of America; Tohoku University, JAPAN

## Abstract

Macrophages play a crucial role in inflammation, a defense mechanism of the innate immune system. Metabolic function powered by glucose transporter isoform 1 (Glut1) is necessary for macrophage activity during inflammation. The present study investigated the roles of cystathionine-γ-lyase (CSE) and its byproduct, hydrogen sulfide (H_2_S), in macrophage glucose metabolism to explore the mechanism by which H_2_S acts as an inflammatory regulator in lipopolysaccharide- (LPS) induced macrophages. Our results demonstrated that LPS-treated macrophages increased Glut1 expression. LPS-induced Glut1 expression is regulated via nuclear factor (NF)-κB activation and is associated with phosphatidylinositol-3-kinase PI3k activation. Small interfering (si) RNA-mediated silencing of CSE decreased the LPS-induced NF-κB activation and Glut1 expression, suggesting a role for H_2_S in metabolic function in macrophages during pro-inflammatory response. Confoundingly, treatment with GYY4137, an H_2_S-donor molecule, also displayed inhibitory effects upon LPS-induced NF-κB activation and Glut1 expression. Moreover, GYY4137 treatment increased Akt activation, suggesting a role in promoting resolution of inflammation. Our study provides evidence that the source of H_2_S, either endogenous (via CSE) or exogenous (via GYY4137), supports or inhibits the LPS-induced NF-κB activity and Glut1 expression, respectively. Therefore, H_2_S may influence metabolic programming in immune cells to alter glucose substrate availability that impacts the immune response.

## Introduction

Hydrogen sulfide (H_2_S) is an endogenously produced inflammatory mediator increasingly recognized for its role in various inflammatory diseases. H_2_S functions as a signaling molecule at physiologic levels influencing several biological processes [1–4]. H_2_S is produced in mammalian cells from L-cysteine predominately by cystathionine γ-lyase (CSE), cystathionine beta-synthase (CBS), and 3-mercaptopyruvate sulfotransferase (3-MST) [5]. H_2_S is a pro-inflammatory mediator produced by the activity of CSE in macrophages [6, 7], which supports their further activation [8]. Excessive H_2_S is proposed to promote inflammation and tissue damage in an animal model of sepsis [9, 10], and inherited retinal disease [11]. A recent report demonstrates the correlation of higher concentrations of plasma levels of H_2_S with an early inflammatory response in septic patients, suggesting that the early elevated H_2_S concentrations influence substance P levels [12]. Thus, the associated role of H_2_S in inflammatory disease necessitates the elucidation of its role in regulating inflammation in macrophages.

Immune responses triggered by Toll-like receptor (TLR) activity in macrophages stimulate the upregulation of CSE and concomitant production of H_2_S, which requires nuclear factor kappa-light-chain enhancer of B-cells (NF-κB), p38 mitogen-activated protein kinase (MAPK), and extracellular signal-regulated kinase (ERK) signaling pathways [7, 13]. NF-κB is a well-characterized signaling network for regulating inflammation [14]. It has been reported that the NF-κB and PI3k/protein kinase B (Akt) signaling pathway control metabolic function via the regulation of hypoxia-inducible factor (HIF)1α in macrophages [15–18]. Glucose transporter isoform 1 (Glut1) was induced by H_2_S activity to stabilize HIF1α in an *in vitro* study of human macrophages [16], suggesting a role for H_2_S in glucose metabolic function.

Immunity and cellular metabolism are two fundamentally linked systems, and the crosstalk between them regulates the immune function of cells [19]. The glucose transporter (Glut) family is a class of hexose transporters that imports glucose into cells. The most widely expressed glucose transporter in tissue and cells is Glut1—which maintains glucose levels to support cellular energy requirements [20, 21]. Glut1 is the primary rate-limiting glucose transporter in macrophages and a critical regulator of macrophage inflammatory response [18]. Upon macrophage stimulation, Glut1 levels are increased via NF-κB [18] which requires PI3K/Akt activation as well [15]; These pathways then culminate to stabilize HIf1α, which in turn regulates Glut1 expression to induce glycolysis for inflammatory activation [22]. The rapid increase in Glut1 during acute inflammation in macrophages drives increased glucose uptake and utilization to induce a reactive oxygen species (ROS) driven pro-inflammatory response [18].

In this study, we investigated the CSE/H_2_S system’s role in macrophage metabolic function during the immune response and examined the role of NF-κB and Akt in this process. We hypothesized that CSE/H_2_S system plays a critical role in inflammation-induced glucose metabolism due to previous evidence that crosstalk exists between H_2_S and the inflammatory NF-κB activities [8]. Our study provides evidence that this system regulates the expression of Glut1 and regulates glucose metabolism in macrophages to influence the immune response. Our study provides evidence that endogenous CSE-derived H_2_S supports the pro-inflammatory expression of Glut1. We also show that exogenous H_2_S from H_2_S-donor molecules reduced LPS-induced Glut1.

## Materials and methods

### Macrophage cell culture

RAW264.7 (ATCC, Manassas, VA) murine macrophage cell lines were cultured in DMEM (Gibco, Waltham, MA) containing 10% (v/v) heat-inactivated fetal bovine serum (FBS; Cell Applications Inc., San Diego, CA), 100 units/ml penicillin, and 100 μg/ml streptomycin (Gibco) and maintained at 37°C in a humidified atmosphere containing 5% CO_2_. Cells were counted to seed 2.4 x 10^6^ cells on 6-well culture plate and grown to confluence. After reaching 70% confluence, macrophages were ready for treatment.

### Macrophage treatment with lipopolysaccharide (LPS)

Macrophages were stimulated with *E*. *coli*-derived LPS (100ng/mL; Invitrogen, Waltham, MA) for 4 h. The concentration of LPS has been reported by other laboratories to induce immune and pro-inflammatory responses in macrophages [7, 8, 13, 23]. After treatment, macrophages were harvested for the preparation of RNA and protein analysis.

### Protein extraction and Western blot

The treated macrophage cells were washed with ice-cold PBS and then lysed in RIPA cell lysis buffer, and 1% Halt protease inhibitor cocktail (Thermo Fisher). The resulting cell lysates were centrifuged for 20 min. at 20,000 g at 4°C, and the protein concentrations in the supernatants were determined using a Pierce BCA protein assay kit (Thermo Fisher) [24]. 20 μg proteins were loaded onto 10% SDS-PAGE gels, followed by electro-transfer onto nitrocellulose-membrane (Bio-Rad). The membranes were blocked in 1 × TBST (0.1% Tween-20, 20 mM Tris–Cl (pH 8.0), and 150 mM NaCl) containing 5% nonfat dry milk powder and then incubated with the primary antibodies against Glut1 (1:1000 dilution, 66290, Proteintech), GAPDH (1:1000 dilution, 60004, Proteintech), overnight at 4°C. Membranes were washed 3 times (1xTBST), and incubated with horseradish peroxidase-conjugated secondary antibodies (1:10000, SA00001, Proteintech) for 1 h at room temperature and then washed 3 times (1 × TBST). Lastly, immunoreactive proteins were detected using enhanced chemiluminescence detection kit (Bio-Rad). The band density was quantified by Image J 1.8.0172 software (National Institutes of Health) and the representative data were experiment normalized to nontreated control.

### RNA extraction and RT-qPCR

Total RNA from cells was extracted using Trizol and chloroform reagents (Invitrogen) following the manufacturer’s instructions. Sample concentrations were determined using Nanodrop One. First-strand cDNA synthesis was performed on 5μg total RNA using M-MLV reverse transcriptase (Invitrogen) and random hexamers (IDT, Coralville, IA) and stored at −20°C. PowerUp SYBR Green Mix (Applied Biosystems, Waltham, MA) was used according to the manufacturer’s instructions in a 384-well format. To compare the mRNA levels between different samples, the 2^-ΔCt^ [25] method was used; and data were normalized to GAPDH. Experiments were run in triplicate; each sample represents three technical repeats. The sense and antisense primers were designed using the PrimerQuest Tool from Integrated DNA Technologies (Table 1).

**Table 1:** PCR primer sequences.

Gene	Forward (5′–3′)	Reverse (5′–3′)
Glut1	GATCTGAGCTACGGGGTCTT	TGTAGAACTCCTCAATAACCTTCTG
CSE	CAAAGCAACACCTCGCACTC	ATGCAAAGGCCAAACTGTGC
GAPDH	CGTCCCGTAGACAAAATGGT	GAGGTCAATGAAGGGGTC

### siRNA-mediated knockdown of CSE gene

Silencer Select pre-designed siRNAs (Ambion, Austin, TX) targeting the CSE gene and negative scramble control siRNA were used in the gene silencing experiments. CSE is the dominant H_2_S-producing enzyme in several tissues, including macrophage [4, 26]; silencing CSE diminishes macrophage capacity to produce endogenous H_2_S. Following manufacturer instructions for lipofectamine RNAiMAX (Invitrogen), cells were incubated with 5pmol siRNA-lipofectamine complex for 24 h. After incubation, the medium was replaced, and cells were further treated.

### NF-κB activity

The commercial kit NF-κB phospho-p65 InstantOne ELISA (eBioscience, 85-86083-11) was used to detect phosphorylated NF-κB in whole cell lysates following the manufacturer’s instructions. Briefly, using 96-well plates, 50μL cell lysates were mixed with 50μL capture antibody cocktail and incubated at room temperature for 1 h. Each well was washed 3 times. Then, 100μL detection reagent was added to each well for 30 min. Stop solution was added, and plate readings were performed immediately at 450 nm.

### Akt activity

The commercial kit Akt (phosphor) pSer473 InstantOne ELISA (eBioscience, 85-86042-11) was used to detect activated (phosphorylated) Akt in whole cell lysates. Using 96-well plates, 100μL cell lysates were added to each well and incubated at room temperature for 2 h. Each well was washed 4 times. Then, 100μL detection reagent was added to each well for 30 min. Stop solution was added, and plate readings were performed immediately at 450 nm. Plate readings were performed at 450 nm. Akt activity was measured following 1 h treatments.

### NF-κB inhibition and PI3K inhibition

RAW 264.7 macrophages were treated with 15μM Bay11-7082 (Cayman Chemical), an inhibitor of κB kinase phosphorylation, for 1 h to inhibit the NF-κB signaling pathway, and then cells were further treated [27]. RAW 264.7 macrophages were treated with 25μM LY294002 (Cayman Chemical), an inhibitor of PI3K activation [28], for 1 h, and cells were further treated. Inhibitors were administered prior to LPS and GYY4137 treatments; fresh media was applied following inhibition.

### Flow cytometry to measure Glut1 surface levels

For immunofluorescence surface staining of macrophages, cells were suspended and fixed at a concentration of 10^6^ cells per 100μL in 4% paraformaldehyde (PFA) for 15 min at room temperature. The cells were then washed with 1X PBS (3 times) and suspended (10^6^ cells per 100μL) in antibody dilution buffer (1X PBS containing 3% bovine serum albumin) with primary antibodies targeting Glut1 for 1 h at 4°C. After that the cells were washed 3 times with 1X PBS followed by incubation with R-phycoerythrin R-PE (Invitrogen), conjugated secondary antibody for 1 h at 4°C. Finally, the cells were washed 3 times with 1X PBS and resuspended in 1X PBS containing 3% bovine serum albumin and read by flow cytometry (Guava MUSE cell analyzer). Data files were analyzed for mean fluorescence intensity by floreada.io software.

### Glutathione level assay

GSH level in macrophages was determined using a one-step fluorometric kit (Fluorometric-Green, ab138881, Abcam) according to the manufacturer’s protocol. Potassium phosphate EDTA buffer (KPE) was prepared freshly, immediately prior to experiments [29]. Following treatments, macrophages were removed from culture plates and counted to 10^6^ cells per aliquot. Aliquoted cells were lysed in 0.5mL KPE buffer containing 0.1% Triton X-100 & 0.6% sulfosalicylic acid and kept on ice. Cell lysates were then vortexed for 15 seconds and centrifuged at 8,000 g for 10 minutes. The supernatants were transferred to pre-chilled microcentrifuge tubes. The concentration of total protein in each sample was determined by Pierce BCA protein assay kit. Samples were then mixed 1:1 with glutathione detection reagent to a final volume of 100μL on 96-well plates and incubated in the dark at room temperature for 30 min. Then, fluorescence intensity was monitored at EX/EM of 490/520 nm. GSH was calculated from the standard curve and reported as GSH * mg protein^-1^.

### Statistical analysis

Statistical differences between experimental groups were determined using statistics software within GraphPad Prism (GraphPad Software, Inc., La Jolla, CA). For all experiments, results are reported for at least n = 3. Data are expressed as mean ± SD. Statistical significance was determined for groups of 3 or more by 1-way analysis of variance (ANOVA) and Tukey’s multiple comparisons. For groups of 2, statistical significance was determined by unpaired Student’s t-test. P ≤ 0.05 was considered statistically significant.

## Results

### LPS induces Glut1 expression in 264.7 macrophages

**Fig 1:**
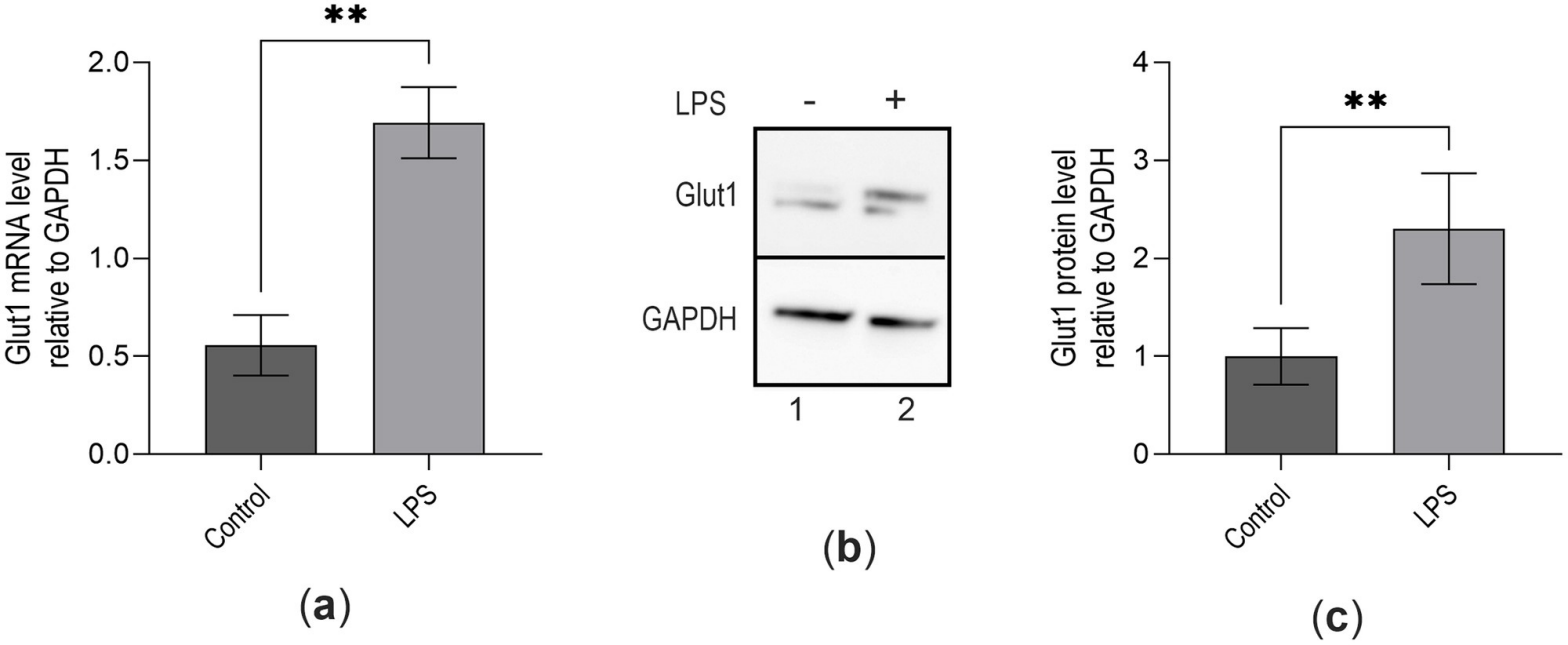
LPS induces Glut1 expression in macrophages. (**a**–**c**) RAW264.7 cells were treated with LPS (100 ng/mL) for 4 h, total RNA was isolated, reverse transcribed to cDNA, and RT-qPCR analyzed the expression Glut1. GAPDH was used as a control. The expression (relative to GAPDH) of Glut1 mRNA is shown in panel (**a**). Protein was extracted from macrophages and analyzed by Western blot using antibodies against Glut1 and GAPDH (loading control) (**b**). Quantifications (using ImageJ software) are shown in panel (**c**). Each experiment was repeated at least with three parallel replicates. Data represent mean ± SD (n = 3); **p < 0.001, ***p < 0.0001.

Studies have demonstrated that H_2_S plays roles in inflammation and elicited a biphasic effect on pro-inflammatory NF-κB activation depending on its source and concentration [8, 23]. NF-κB gene regulatory activities increase Glut1 gene transcription in macrophages during LPS stimulation to fuel the inflammatory response [18, 30]. Due to the potential crosstalk of H_2_S with NF-κB activation, we investigated whether H_2_S plays any role in NF-κB-mediated Glut1 expression. RAW264.7 macrophages were treated with LPS, and Glut1 expression was measured. Briefly, RNA from the control and LPS-treated macrophages were reverse-transcribed into cDNA and analyzed by qPCR using primers specific to Glut1. The expression of Glut1 is significantly increased by 2.4-fold (1.70±0.18 vs. 0.56±0.15; p<0.001) by LPS treatment vs. control (Fig 1a). Western blot analysis also confirms that Glut1 protein expression significantly increases by 2.16-fold (p<0.001) following treatment with LPS (Fig 1b, quantification in Fig 1c). These observations show that Glut1 is upregulated upon LPS-induced inflammation and is potentially involved in glucose uptake and metabolism during inflammation in macrophages.

### NF-κB and PI3k regulate glut1 expression during LPS stimulation

**Fig 2:**
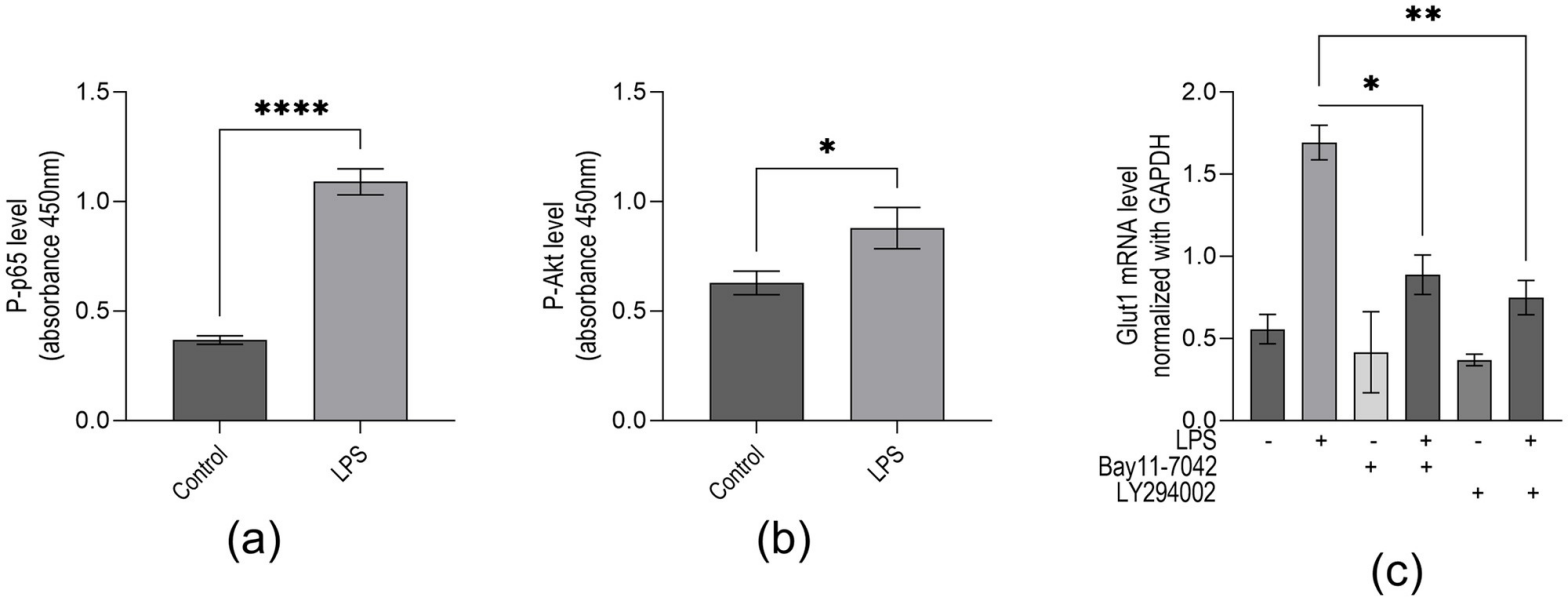
LPS induces phosphorylation of NF-κB subunit p65 and phosphorylation of Akt subunit ser473, and NF-κB and PI3k are associated with Glut1 expression in macrophages. (**a**–**c**) RAW264.7 cells were initially treated with IKK-inhibitor Bay11-7042 (15μM) or PI3k inhibitor LY7294002 (25μM) for 1 h and then treated with LPS for an additional 4 h for RNA analysis. ELISA analyzed proteins by targeting phospho-p65 (NF-κB subunit) (panel **a**). Akt proteins were analyzed by ELISA targeting Phospho-Ser473 (Akt subunit) (panel **b**). (**c**) RNA was isolated from the above treated and control cells. RT-qPCR measured expressions (relative to GAPDH) of Glut1. GAPDH was used as a control. Data represent mean ± SD (n = 3); *p < 0.05, **p < 0.001, ****p < 0.00001.

The transcription factor NF-κB activation plays a central role in inflammation and immune response. Without inflammatory stimulus, NF-κB is complexed with IκBα and remains inactive. LPS interaction with TLR-4 stimulates a phosphorylation cascade leading to IκBα degradation and NF-κB translocation to the nucleus, activating the transcription of target genes [31]. To investigate if NF-κB activation is associated with LPS-induced Glut1 expression, we treated macrophages with an irreversible IKK kinase inhibitor (Bay11-7042) and analyzed its impacts on LPS-induced Glut1 expression. ELISA analysis demonstrates that upon treatment with LPS, the level of phospho-p65 (NF-κB subunit) is increased 2.94-fold (0.37±0.02 vs. 1.09±0.06; p<0.001) (Fig 2a). Studies have demonstrated that inflammatory signaling promotes glucose uptake via PI3k/Akt regulation of Glut1 activity and trafficking [32, 33]. Here, our ELISA analysis demonstrates that LPS induces phospho-Akt (Akt Ser473 subunit) significantly 1.4-fold (0.63±0.05 vs. 0.88 SD±0.09; p<0.0252) (Fig 2b). To determine if PI3K pathway activation is associated with increased LPS-induced Glut1 expression, we treated cells with an inhibitor of PI3K (LY294002) and analyzed its effect on LPS-induced Glut1 expression. The LPS-induced increase in Glut1 expression is significantly decreased by treatment with Bay11-7042 (0.89±0.21 vs. 1.70±0.18; p<0.011). In addition, LY294002 significantly decreased LPS-induce Glut1 expression (0.75±0.18 vs. 1.70±0.18; p<0.001) (Fig 2c). Thus intact PI3k signaling is necessary for LPS-induced Glut1 expression. These observations suggest that LPS treatment resulted in NF-κB and Akt activation and subsequent Glut1 expression; inhibiting these pathways reduced this expression.

### CSE regulates LPS-induced Glut1 expression

**Fig 3:**
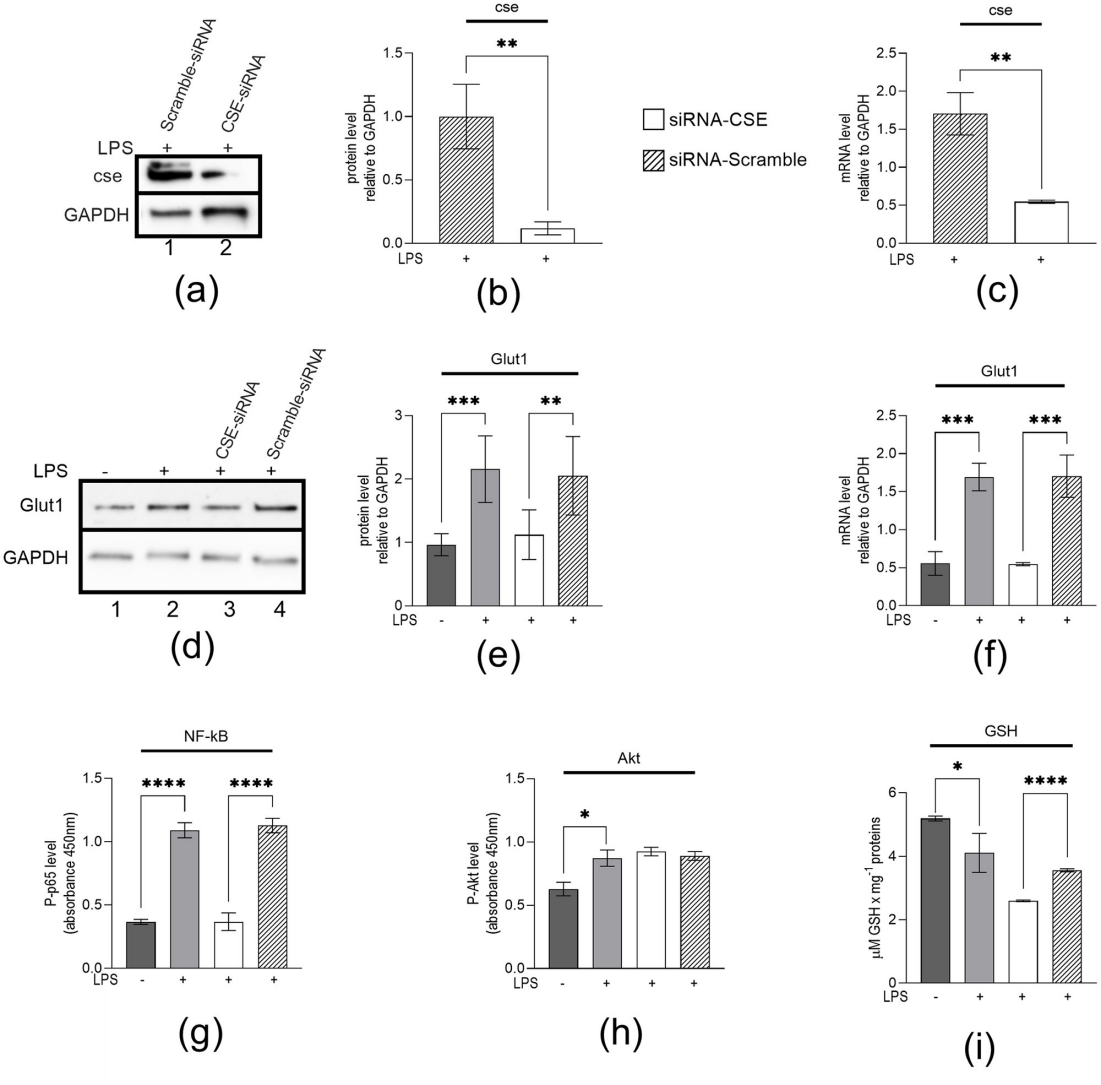
LPS stimulated macrophages genetically silenced of CSE gene to inhibit CSE expression, decreased NF-κB activation (Phospho-p65), Glut1 expression, and glutathione (GSH) level. (**a**–**f**) RAW264.7 cells were transfected (24 h) with CSE-siRNA or Scramble-siRNA, followed by treatment with LPS. RAW264.7 cells were analyzed by Western blotting using antibodies against CSE, Glut1, and GAPDH (loading control) (panels **a** and **d**). The changes in amounts of Glut1 have been quantified by ImageJ software and shown in panels (**b** and **e**). (Panel **c** and **f**) RT-qPCR analyzed RNA for the expression of CSE and Glut1. GAPDH was used as a control. RAW 264.7 cells were analyzed by ELISA targeting Phospho-p65 (NF-κB subunit) (panel **g**) or Phospho-Ser473 (Akt subunit) (panel **h**). GSH levels were determined by a fluorometric kit. Samples were mixed with reagent, and fluorescence intensity was monitored at EX/EM of 490/520 nm. GSH was calculated from the standard curve and reported as GSH * mg protein-1. Data represent mean ± SD (n = 3); *p < 0.05, **p < 0.001, ***p < 0.0001, ****p < 0.00001.

It was reported that CSE plays a key role in NF-κB activation [8, 13]. Here, to investigate if CSE plays any role in regulating LPS-induced Glut1 expression, glucose uptake, and metabolism, we knocked down CSE (using siRNA) in macrophages, then treated them with LPS, and analyzed its impacts on Glut1 RNA and protein expression. Briefly, RAW264.7 macrophages were transfected with CSE or scramble siRNAs (24 h) and treated with LPS (4 h). RNA and proteins were isolated and analyzed by RT-qPCR and Western blot. The expression of CSE is significantly decreased at the protein (0.12±0.05 vs. 1±0.25; p = 0.004) and mRNA (0.55±0.02 vs. 1.70±0.28; p = 0.002) levels upon application of CSE siRNA (Fig 3a–3c). Western blot analysis showed that LPS-induced Glut1 protein level is significantly decreased upon CSE-knockdown (1.22±0.39 vs. 2.16±0.53; p = 0.006) (CSE-siRNA treatment, Fig 3d, quantification in Fig 3e). RT-qPCR analysis showed that LPS-induced Glut1 expression (mRNA level) is significantly decreased upon CSE-knockdown (0.55±0.02 vs. 1.71±0.28; p = 0.002) (Fig 3f). ELISA analysis also showed that LPS-induced Phospho-p65 levels are reduced upon CSE knockdown (0.37±0.07 vs. 1.13±0.06; p<0.001) (Fig 3g). However, the Phospho-s473 (Akt Ser473 subunit) level did not change under CSE-knockdown conditions (Fig 3h). Therefore, the CSE/H_2_S system supports pro-inflammatory NF-κB activity and glucose metabolism.

Previously, it was shown that inhibition of the CSE/H_2_S system dysregulated glutathione (GSH) status, and this could be partially ameliorated with H_2_S donor molecules [34]. Here, we show that the level of GSH was significantly decreased following LPS treatment (4.11±0.62 vs. 5.19±0.08; p = 0.039). However, consistent with previous reports, CSE-knockdown conditions significantly decreased GSH levels below the scramble siRNA group in LPS-treated cells (2.60±0.02 vs. 3.56±0.05 μM GSH per mg protein; p<0.001) (Fig 3i). These results demonstrate the important role of CSE and H_2_S in regulating GSH levels in macrophages.

### H_2_S regulates LPS-induced Glut1 expression

**Fig 4:**
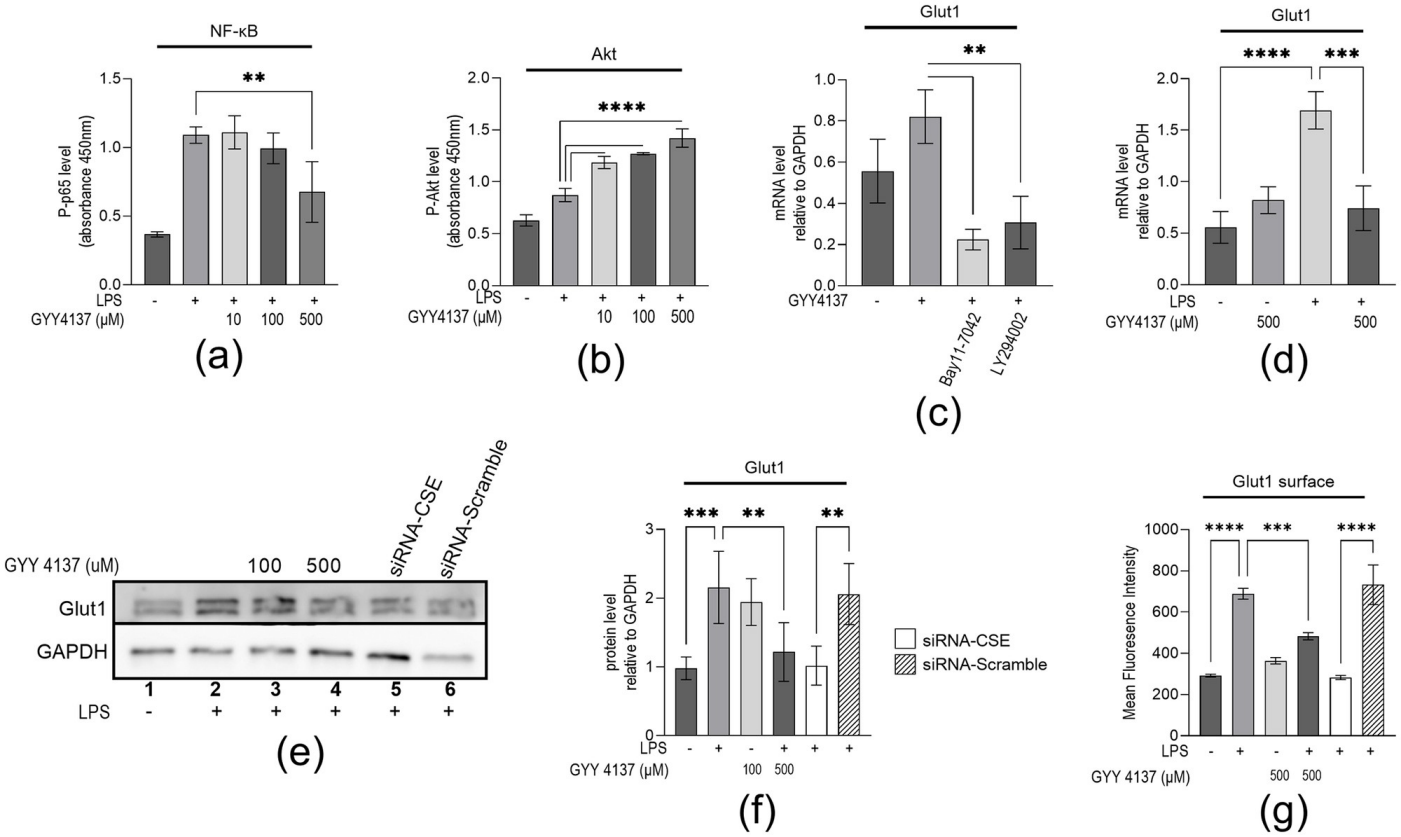
H_2_S decreased LPS-induced NF-κB activation and increased Akt activity which is associated with Glut1 mRNA, protein, and surface expression on macrophages. (**a**–**b**) RAW264.7 cells were treated simultaneously with indicated concentrations of GYY4137 and LPS. (Panel **a** and **b**) RAW 264.7 cells were analyzed by ELISA targeting Phospho-p65 (NF-κB subunit) (panel **a**) or Phospho-Ser473 (Akt subunit) (panel **b**). (**c**) RAW264.7 cells were initially treated with IKK-inhibitor Bay11-7042 (15μM) or PI3k inhibitor LY7294002 (25μM) (for 1 h) and then treated with 500μM GYY4137 for an additional 4 h for RNA. RT-qPCR analyzed RNA for the expression of Glut1 (panel **c**). GAPDH was used as a control. (**d**) RAW264.7 cells were treated with LPS and 500μM GYY4137 for 4 h for RNA. RT-qPCR analyzed RNA for the expression of Glut1 (panel **d**). GAPDH was used as a control. (**e**—**f**) RAW264.7 cells were transfected (24 h) with CSE-siRNA or Scramble-siRNA, followed by treatment with GYY4137 and LPS for 4 h. RAW264.7 cells were analyzed by Western blotting using antibodies against Glut1 and GAPDH (loading control) (panel **e**). The changes in amounts of Glut1 have been quantified by ImageJ software and shown in panel (**f**). (**g)** RAW264.7 cells were transfected (24 h) with CSE-siRNA or Scramble-siRNA, followed by treatment with GYY4137 and LPS for 4 h. RAW264.7 cells surface staining were analyzed by flow cytometry using antibodies against Glut1 and secondary antibodies conjugated with R-PE (565/590 nm). Data represent mean ± SD (n = 3); **p < 0.001, ***p < 0.0001, ****p < 0.00001.

Previously, Lohninger *et al*. (2015) reported that treatment with the H_2_S-donor molecule, GYY4137, induced the expression of Glut1 by stabilizing HIF1α under normal oxygen conditions in THP-1 macrophages [16]. This same study also reported that high concentrations of H_2_S decreased NF-κB activation, which is consistent with another group’s report on RAW264.7 macrophages [23]. Contrary to previous reports, we suggested the roles of endogenous H_2_S to support pro-inflammatory Glut1 expression within the CSE/H_2_S system in the previous section. However, exogenous H_2_S regulating inflammation-induced Glut1 expression in macrophages is poorly understood. Here, to investigate if H_2_S plays any role in LPS-induced Glut1 expression, we treated LPS-stimulated macrophages with either 10, 100, or 500μM GYY4137 for 4h simultaneous to LPS being administered; these concentrations achieve a steady state concentration of around 0.32, 3.2, and 16 μmol/L of H_2_S for an over 24-hour period, respectively [16]. The impacts of H_2_S levels on NF-κB activation, Akt activation, Glut1 RNA, and protein expression were measured. Briefly, RAW264.7 macrophages were treated with LPS simultaneous with the indicated concentration of GYY4137 for 4h. Initially, we sought to elucidate the effect of H_2_S on key pathways that regulate inflammatory glucose uptake and metabolic function (NF-κB and Akt pathways). ELISA analysis showed that in LPS-treated macrophages also treated with 500μM GYY4137, Phospho-p65 levels were significantly decreased compared to the 0μM group (0.68±0.22 vs. 1.09±0.06; p<0.001) (Fig 4a). Interestingly, all tested concentrations of GYY4137 significantly increased Phospho-Ser473 (Akt subunit) levels vs LPS alone (10μM– 1.19±0.06; 100μM– 1.27±0.01; 500μM– 1.42±0.09 vs. 0.87±0.06; p<0.001) (Fig 4b).

RNA and proteins were isolated and analyzed by RT-qPCR and Western blot, respectively. Our RT-qPCR analysis demonstrated that the expression of Glut1 increased but not significantly (p = 0.086) upon treatment with 500μM GYY4137, without the addition of LPS. This expression of Glut1 is significantly reduced upon application of IKK-inhibitor, Bay11-7042 (0.22±0.05 vs. 0.82±0.13; p = 0.0018), or PI3K inhibitor, LY294002 (0.31±0.13 vs. 0.82±0.13; p = 0.008) (Fig 4c). RT-qPCR analysis showed that LPS-induced Glut1 is decreased upon simultaneous treatment of LPS with 500μM GYY4137 (0.74±0.15 vs. 1.70±0.18, p = 0.002) (Fig 4d). LPS increased Glut1 protein level, on Western is decreased with treatment of 500μM GYY4137 (1.22±0.43 vs. 2.17±0.53; p<0.0082) (compare lanes 2 and 4) (Fig 4e, quantification in Fig 4f). Interestingly, Glut1 protein level with the application of siRNA targeting CSE group is similarly decreased compared to scramble siRNA (1.02± 0.28 vs. 2.06± 0.44; p<0.001) (compare lanes 5, and 6) (Fig 4e, quantification in Fig 4f).

To understand the function of H_2_S in Glut1, the surface expression of Glut1 on macrophages was measured by flow cytometry. We silenced CSE in RAW264.7 cells by siRNA and scramble-siRNA and then treated with LPS without or with 500μM GYY4137. The cells were then stained with Glut1 antibody followed by R-phycoerythrin-conjugated secondary antibody. Cells were then analyzed by flow (MUSE cell analyzer). We observed that Glut1 expression levels were low in the control cells (mean fluorescence intensity MFI = 291±13.6) and significantly increased upon LPS stimulation (689±26.3; p<0.001) (Fig 4g). Interestingly, upon CSE-knockdown (CSE-siRNA and LPS treatments), the level of LPS-induced Glut1 expression was decreased relative to scramble siRNA and LPS (282.7±10.1 vs. 732.6±96.1). The surface level of LPS-induced Glut1 was similarly decreased following treatment with GYY4173 (GYY4137 and LPS treatments). Scramble siRNA has no significant impact on LPS-induced expression of Glut1. These results further support our observation that endogenous H_2_S is required for LPS-induced Glut1 in macrophages and that high levels of H_2_S negatively impact this expression.

## Discussion

Inflammation is a biological response of the immune system triggered by various factors, including microbial invaders and injury [35], that induces the coordinated activation of signaling pathways regulating inflammatory mediators and immune cells [36]. This response is metabolically expensive and is fueled primarily by glucose metabolism [37]. Glut1 is the primary rate-limiting glucose transporter in macrophages and a critical regulator of macrophage inflammatory response [18, 30]. Studies in the human THP-1 macrophage cell line revealed that H_2_S induced the expression of Glut1 and decreased its pro-inflammatory effect [16], suggesting the potential role of H_2_S and H_2_S-producing enzymes in inflammatory glucose metabolism in macrophages. Thus, the CSE/H_2_S system’s role in Glut1 metabolism may be a relevant target for controlling macrophage inflammation.

Based on the evidence of crosstalk between H_2_S and macrophage metabolism [16], we posited that the CSE/H_2_S system is involved in inflammation-induced metabolism in macrophages. We confirmed the expression of CSE and demonstrated that the inflammatory Glut1 expression is induced in macrophages upon LPS stimulation, in accordance with previous studies [16]. CSE expression in macrophages is well known to play a significant role in H_2_S generation during the immune response [7–9]. However, the role of CBS for the generation of H_2_S in macrophages is reported to be negligible during an inflammatory response and therefore was not investigated here [38–40]. CSE expression in macrophages highlights these systems’ importance in macrophage biological activities.

NF-κB plays a central role in inflammatory signaling by regulating the transcription of many pro-inflammatory cytokines, chemokines, and inflammatory mediators [41]. We demonstrated that the inhibition of IKK by Bay11-7042 significantly reduces LPS-induced Glut1 expression, implicating NF-κB in inflammation-induced Glut1 expression. In addition, our results suggest PI3k activity is associated with LPS-induced Glut1 expression. Indeed, inhibition of PI3k activation using the PI3k inhibitor LY294002 reduced LPS-induced Glut1 expression, demonstrating its role in LPS-induced metabolic function in macrophages. The metabolic regulation of activated macrophages converges from NF-kB and Akt activities upon Hif1α, a key regulator of Glut1 expression [42]. Our Nf-kB and PI3k inhibition studies agree with previous reports indicating that PI3k/Akt and NF-κB activation control macrophage inflammatory metabolism and Glut1 expression [15, 17, 18]. Next, we investigated the role of endogenous and exogenous H_2_S in influencing these pathways following macrophage LPS stimulation.

We demonstrate that knockout of the CSE gene with siRNA decreased NF-κB activation in response to LPS stimulation, which is consistent with a previous report [8]. Glut1 expression is also decreased in LPS-treated macrophages genetically silenced of CSE, suggesting an impairment of the inflammation-induced metabolic program. These observations suggest a role of CSE in LPS-induced Glut1 expression via NF-κB. The interaction between H_2_S and NF-κB is poorly understood. However, modifications via S-sulfhydration of cysteine residues have been suggested elsewhere. Previously, Sen *et al*. (2012) reported that H_2_S modified cysteine residues of the P65 subunit of NF-κB, which enhanced binding to anti-apoptotic genes [43]. Our CSE gene knockout studies demonstrate that CSE plays a role in NF-κB activation and that silencing CSE suppresses NF-κB-DNA binding potential. These results show the importance of the CSE/H_2_S system to support NF-κB activation and the pro-inflammatory function in macrophages. However, additional work is required to elucidate the exact mechanisms that dictate the interactions between endogenous H_2_S produced by CSE and pro-inflammatory NF-κB activity.

Glucose metabolism and signaling involve many upstream regulators and signaling such as PI3k/Akt. In this study, we show that H_2_S from the donor molecule, GYY4137, increases the level of activated Akt in LPS-stimulated cells. Previous studies on THP-1 macrophages have found that Akt activation stabilizes HIf1α to induce Glut1 expression [44]. In lymphoid cell line FL5.12, PI3k activity was reported to regulate Glut1 trafficking and activated Akt was sufficient to maintain glucose uptake and surface Glut1 in the absence of cytokine stimulation [33]. Akt activity was also reported to be critical for the resolution of inflammation and induce anti-inflammatory alternatively activated (M2) macrophages [45]. Indeed, CSE/H_2_S system activity to stabilize nuclear translocation of HIf1α was shown to promote the resolution of inflammation and injury during colitis, and H_2_S donor molecules further enhance this protection [46]. Our data support the role that H_2_S donor molecules promote Akt activity and may serve an anti-inflammatory role. However, Akt activity was not directly associated with Glut1 expression or surface translocation within our study.

We show that the role of the CSE/H_2_S system is implicated in the expression of Glut1 under LPS stimulation via NF κB; we next investigated whether the H_2_S donor molecule, GYY4137, can alter these same systems and Glut1 expression. We show that NF-κB activation is decreased in macrophages upon treatment with 500μM GYY4137 under LPS stimulation. 10μM and 100μM GYY4137 had little effect on NF-κB activation and Glut1 expression. In addition, we show that treatment with 500μM GYY4137 decreased LPS-induced Glut1 expression. Our results show that high levels of H_2_S decrease NF-κB activation, potentially attenuating the macrophage pro-inflammatory response. It was reported that under prolonged exposure (24h) to H_2_S, HIF1α is stabilized, increasing Glut1 expression and decreasing inflammatory activity [16]. Our results support the conclusion that H_2_S donors may serve an anti-inflammatory function due to the attenuation of NF- κB activation.

Though hydrogen sulfide has been considered as a tissue protectant in some pathological conditions, but the mechanisms of tissue protection is a point of controversy. This role of hydrogen sulfide may involve the direct actions of this molecules from the indirect downstream effects. The controversy roles of hydrogen sulfide are particularly important in inflammation research. Several groups have previously reported the anti-inflammatory activity exhibited by H_2_S donor molecules upon NF-κB activity in macrophages [16, 23]. Overall, we provide evidence that CSE/H_2_S system regulates inflammation and serves a role in LPS-induced Glut1 expression. Future studies will be required to elucidate how the flux through various glycolytic pathways, such as the Pentose Phosphate Pathway, is influenced by H_2_S. Indeed, H_2_S influence on ROS is suggested in numerous studies [47–52]. Here, we demonstrate that endogenous CSE/H_2_S are important for GSH production and serve a role in antioxidant defenses. However, absent CSE, GSH levels were markedly decreased but were not associated with increased NF- κB activation within our study parameters The role of the CSE/H_2_S system upon Glut1 expression suggests the potential role played by endogenous H_2_S to support pro-inflammatory flux through ROS-producing glycolytic pathways [18].

Based on our H_2_S-donor experiments and previous studies, we hypothesize that exogenous H_2_S may exert an inhibitory influence on ROS production, inhibiting pro-inflammatory response in macrophages. Additionally, we showed that H_2_S donor treatment increased Akt activation; thus, it is suggested that H_2_S donors exert an anti-inflammatory role. It is necessary to investigate the role of these systems in primary macrophages, as this study is limited to the investigation of linear mouse RAW264.7 cell line. Overall, our observations demonstrate the novel regulatory pathway that H_2_S influences in macrophage immune response.

## Conclusions

Our results show the critical roles of CSE and H_2_S in modulating glucose metabolism in macrophages. The endogenous CSE/H_2_S system supports NF-κB activation and Glut1 expression under LPS-induced inflammation in macrophages. However, contradictorily, H_2_S-donor molecules are demonstrated to inhibit NF-κB and Glut1 in LPS-treated macrophages at high levels. Thus, we show a regulatory network by which H_2_S may influence glucose metabolism and induce metabolic reprogramming in macrophages during inflammation. Inflammation-induced Glut1 expression requires intact NF-κB and PI3k pathways. We show that H_2_S donors enhance Akt activation, a downstream target of PI3k. Both PI3k and NF-κB were demonstrated to be critical for LPS-induced Glut1 expression. Overall, H_2_S and CSE display regulatory roles that may influence the inflammatory potential of macrophages.

## Supporting information

S1 Raw image (PDF)

S2 Raw image (PDF)

S1 Materials (DOCX)
