# Supplementary material for: Cystathionine γ-lyase and hydrogen sulfide modulates glucose transporter Glut1 expression via NF-κB and PI3k/Akt in macrophages during inflammation
Source: PLoS One. 2022 Dec 15;17(12):e0278910. doi: 10.1371/journal.pone.0278910 (PMC9754168; doi:10.1371/journal.pone.0278910)
Supplement: S1 Raw image — (PDF) [file pone.0278910.s001.pdf]

Uncropped Western images

Cystathionine γ-lyase and hydrogen sulfide modulates glucose transporter Glut1 expression via NF-κB and PI3k/Akt in macrophages during inflammation

Alex Cornwell[1], Samantha Fedotova[2], Sara Cowan[2], and Alireza Badiei[2,*]

[1] Department of Biology and Wildlife, College of Natural Science and Mathematics, University of Alaska Fairbanks, AK, USA.
[2] Department of Veterinary Medicine, College of Natural Science and Mathematics, University of Alaska Fairbanks, AK, USA.

* Correspondence: abadiei@alaska.edu

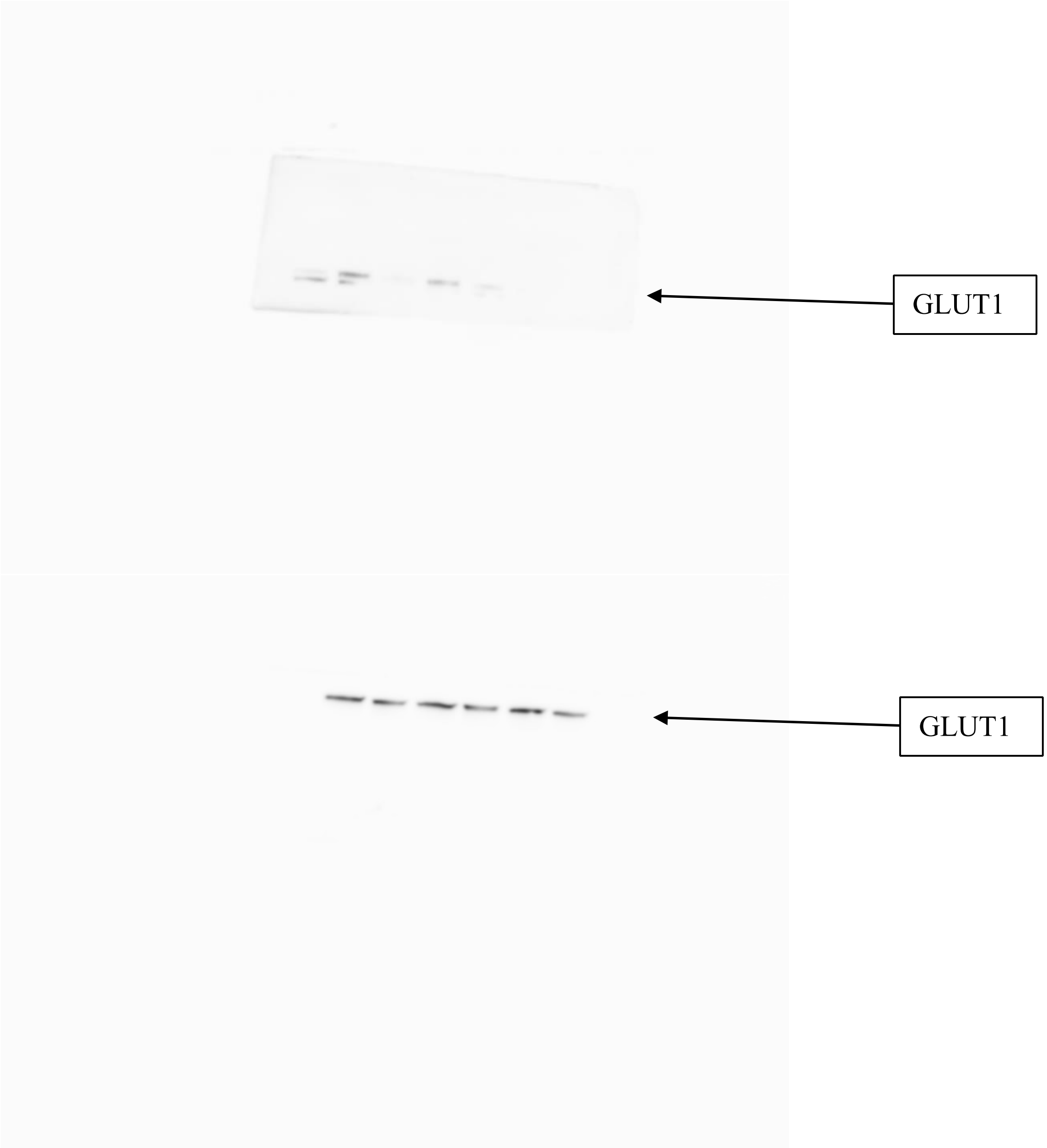

Figure B1. Left to right: Control, LPS, siRNA-CSE, siRNA-CSE+LPS, GYY4137 (0.1mM), and GYY4137 (0.5mM). Underlined samples were not used in final analysis.

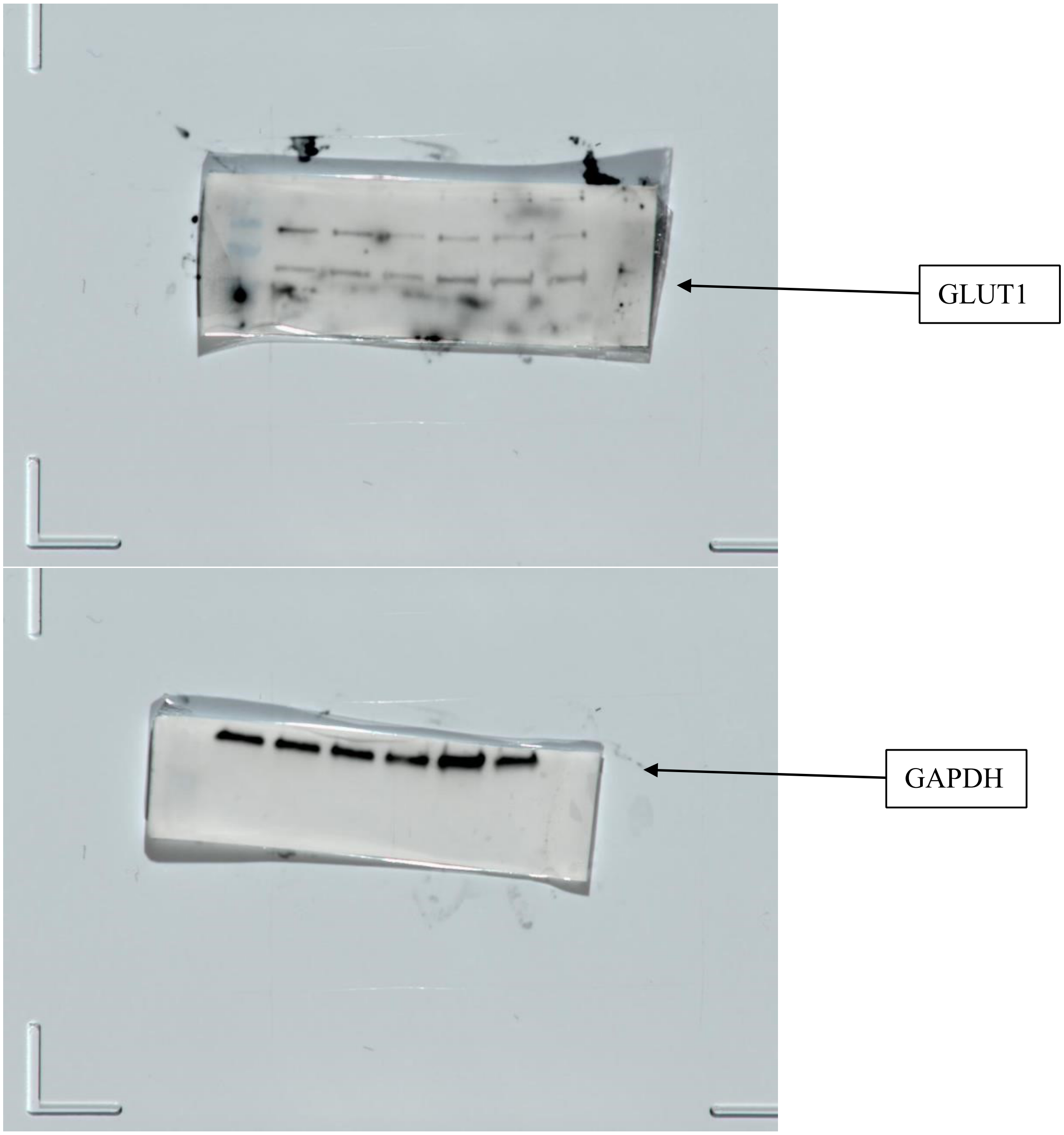

Figure B2. Left to right: Control, LPS, siRNA-CSE+LPS, siRNA-Scramble+LPS, GYY4137 (0.5mM)+LPS, and GYY4137 (0.1mM)+LPS.

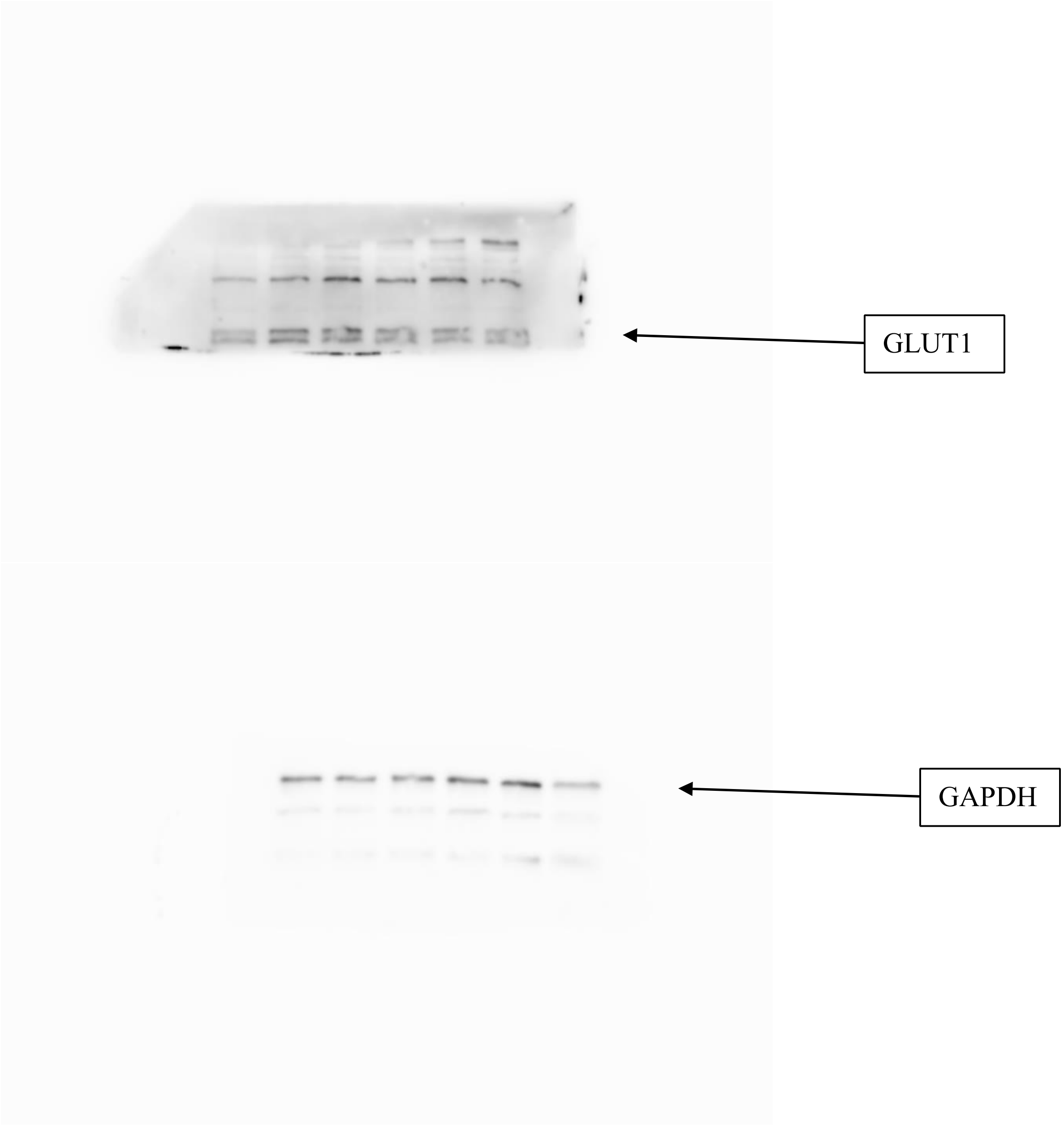

Figure B3. Left to right: Control, LPS, GYY4137 (0.1mM)+LPS, and GYY4137 (0.5mM)+LPS, siRNA-CSE+LPS, siRNA-Scramble+LPS

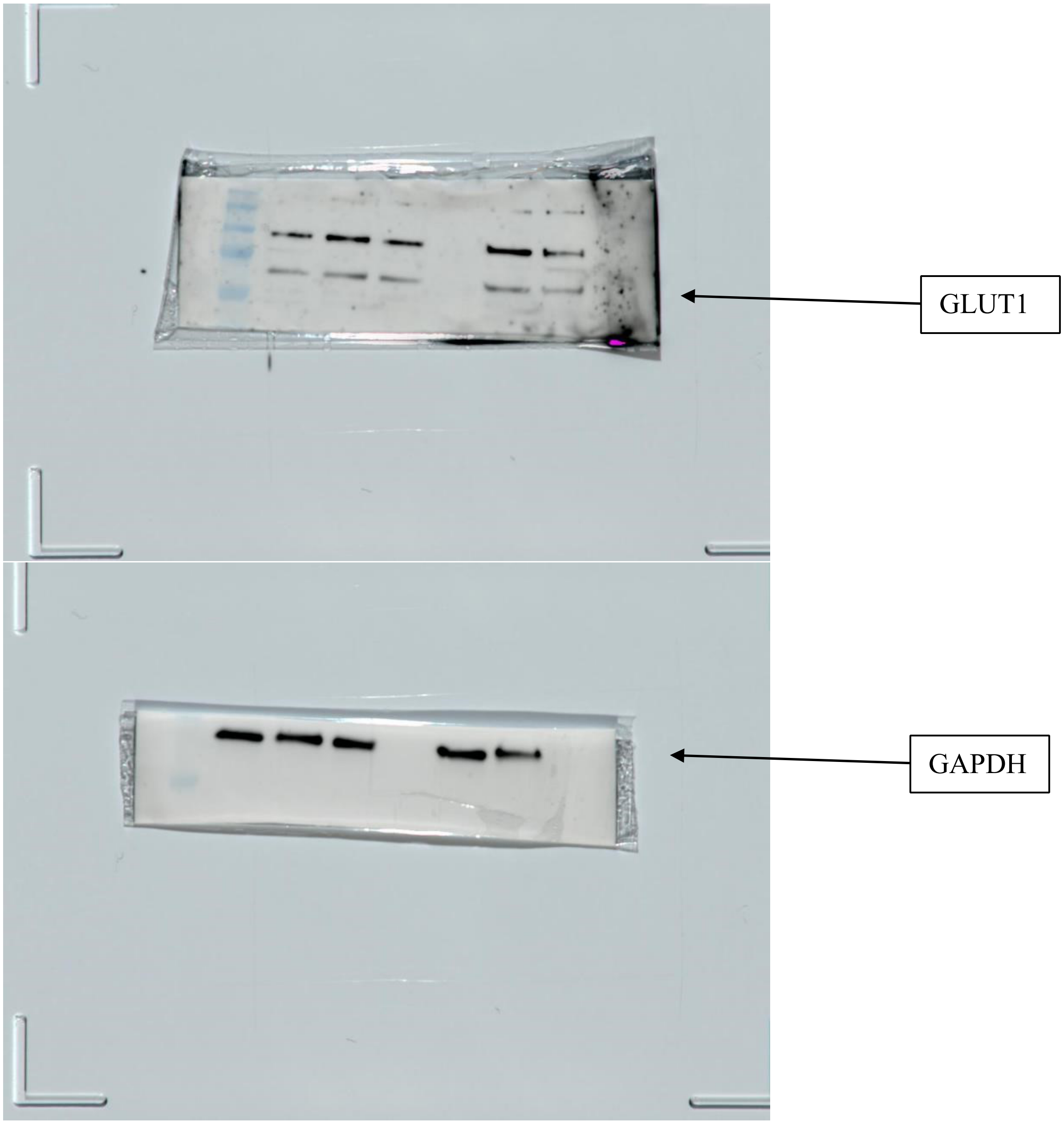

Figure B4. Left to right: Control, LPS, siRNA-CSE+LPS, siRNA-Scramble+LPS, and GYY4137 (0.5mM)+LPS.

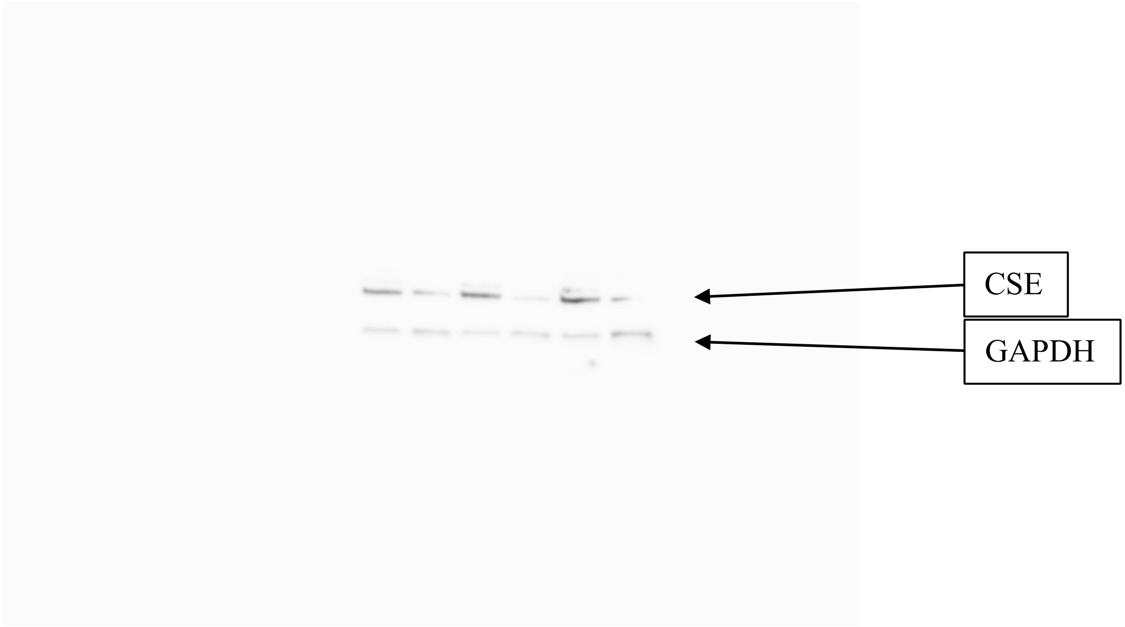

Figure B5. Left to right: siRNA-scramble+LPS, siRNA-CSE+LPS, siRNA-scramble+LPS, siRNA-CSE+LPS, siRNA-scramble+LPS, siRNA-CSE+LPS.

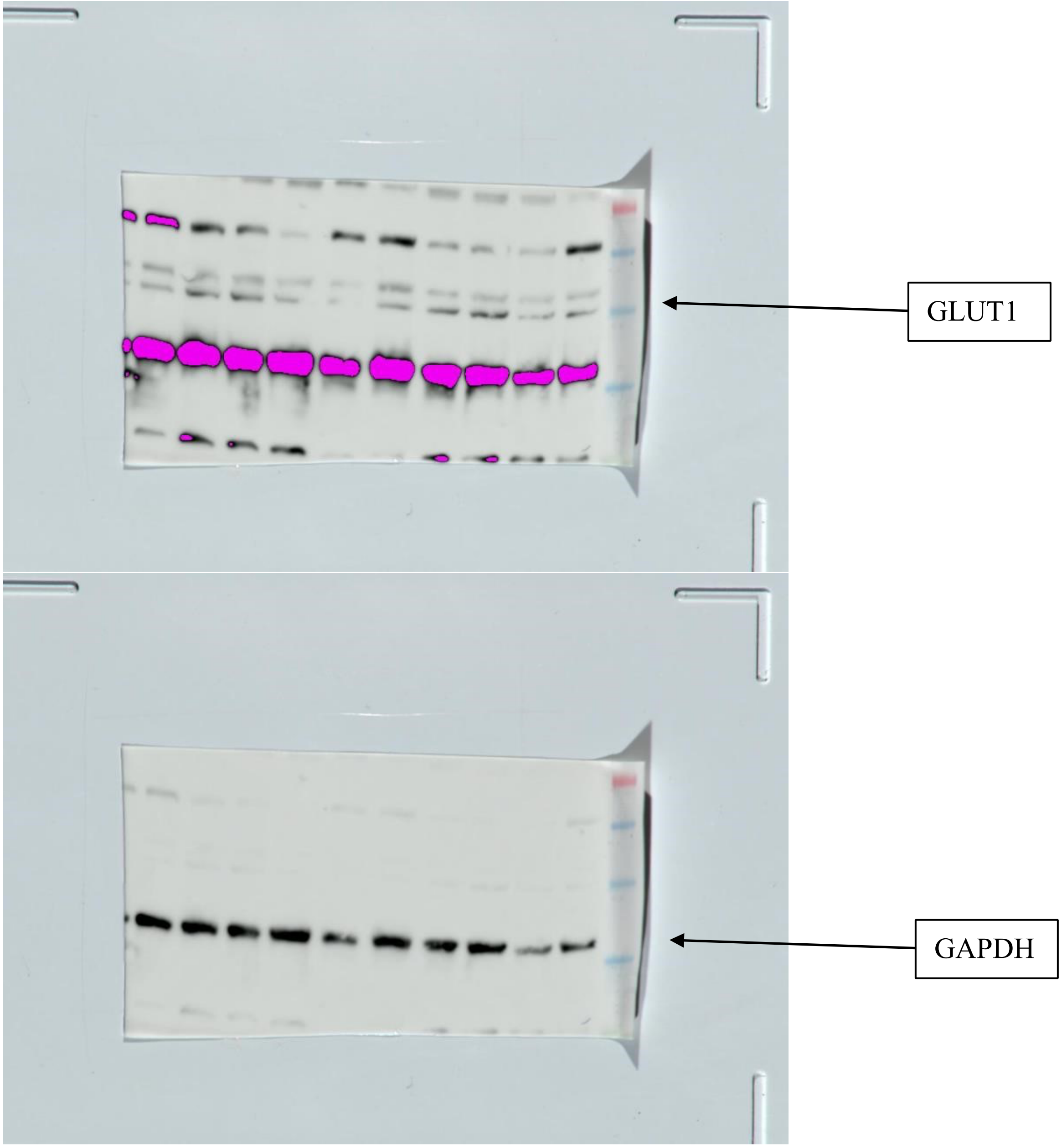

Figure B6. Left to right: Control, LPS, LPS, siRNA-CSE, siRNA-CSE, siRNA-CSE+LPS, siRNA-CSE+LPS, siRNA-CSE+LPS, GYY4137 (0.1mM)+LPS, GYY4137 (0.1mM)+LPS, siRNAScramble+LPS. Underlined samples were not used in final analysis.
