# Supplementary material for: Cystathionine γ-lyase and hydrogen sulfide modulates glucose transporter Glut1 expression via NF-κB and PI3k/Akt in macrophages during inflammation
Source: PLoS One. 2022 Dec 15;17(12):e0278910. doi: 10.1371/journal.pone.0278910 (PMC9754168; doi:10.1371/journal.pone.0278910)
Supplement: S2 Raw image — (PDF) [file pone.0278910.s002.pdf]

# Supplementary information

# Cystathionine γ-lyase and hydrogen sulfide modulates glucose transporter Glut1 expression via NF-κB and PI3k/Akt in macrophages during inflammation

Alex Cornwell[1], Samantha Fedotova[2], Sara Cowan[2], and Alireza Badiei[2,*]

[1] Department of Biology and Wildlife, College of Natural Science and Mathematics, University of Alaska Fairbanks, AK, USA.
[2] Department of Veterinary Medicine, College of Natural Science and Mathematics, University of Alaska Fairbanks, AK, USA.

* Correspondence: abadiei@alaska.edu

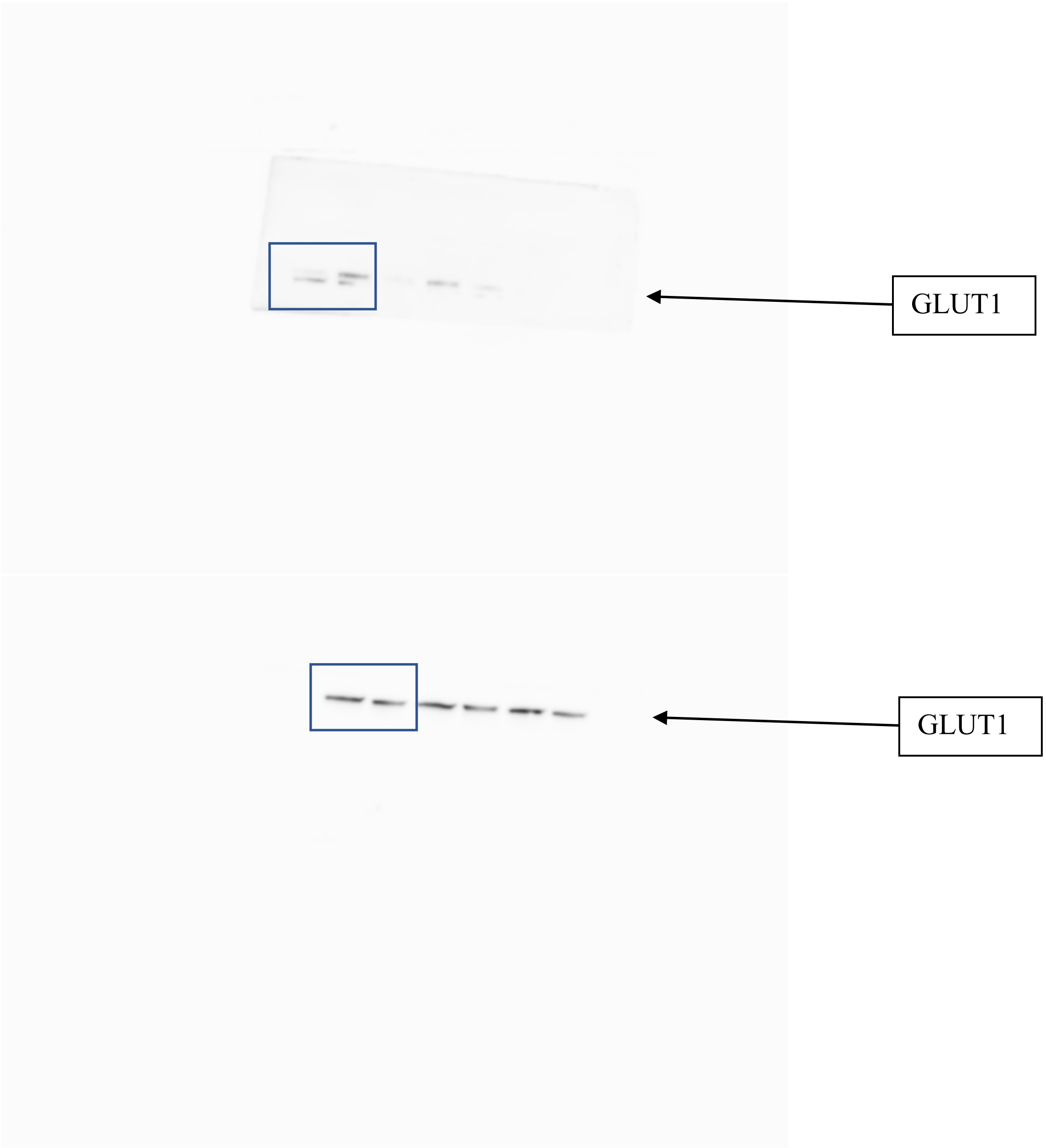


**Figure S1**. LPS induces Glut1 expression in macrophages. Figure 1b was cropped from the above image.

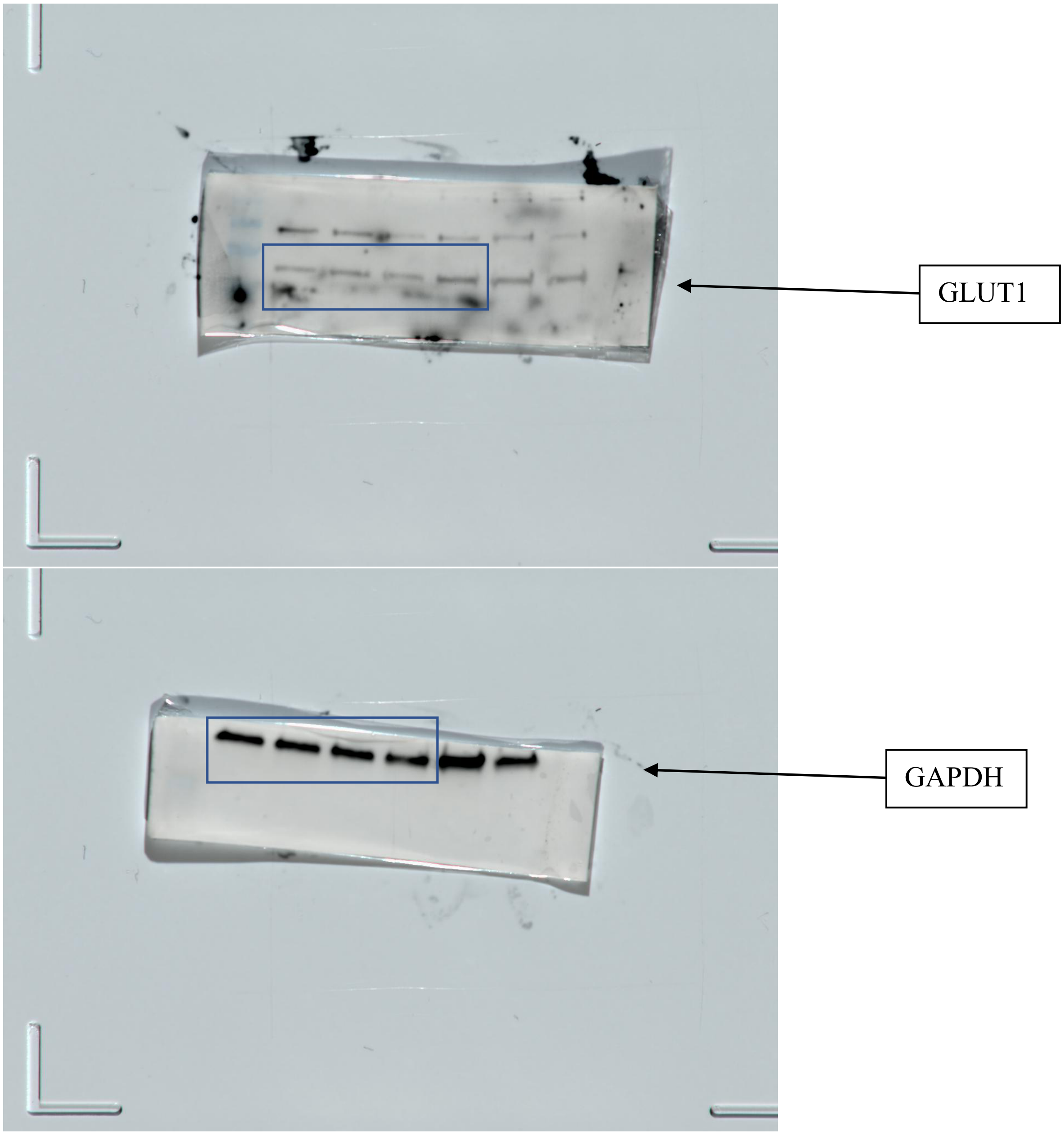


**Figure S2**. LPS stimulated macrophages genetically silenced of CSE gene to inhibit CSE expression decreased Glut1 expression compared to scramble siRNA control. Figure 3b was cropped from the above image.

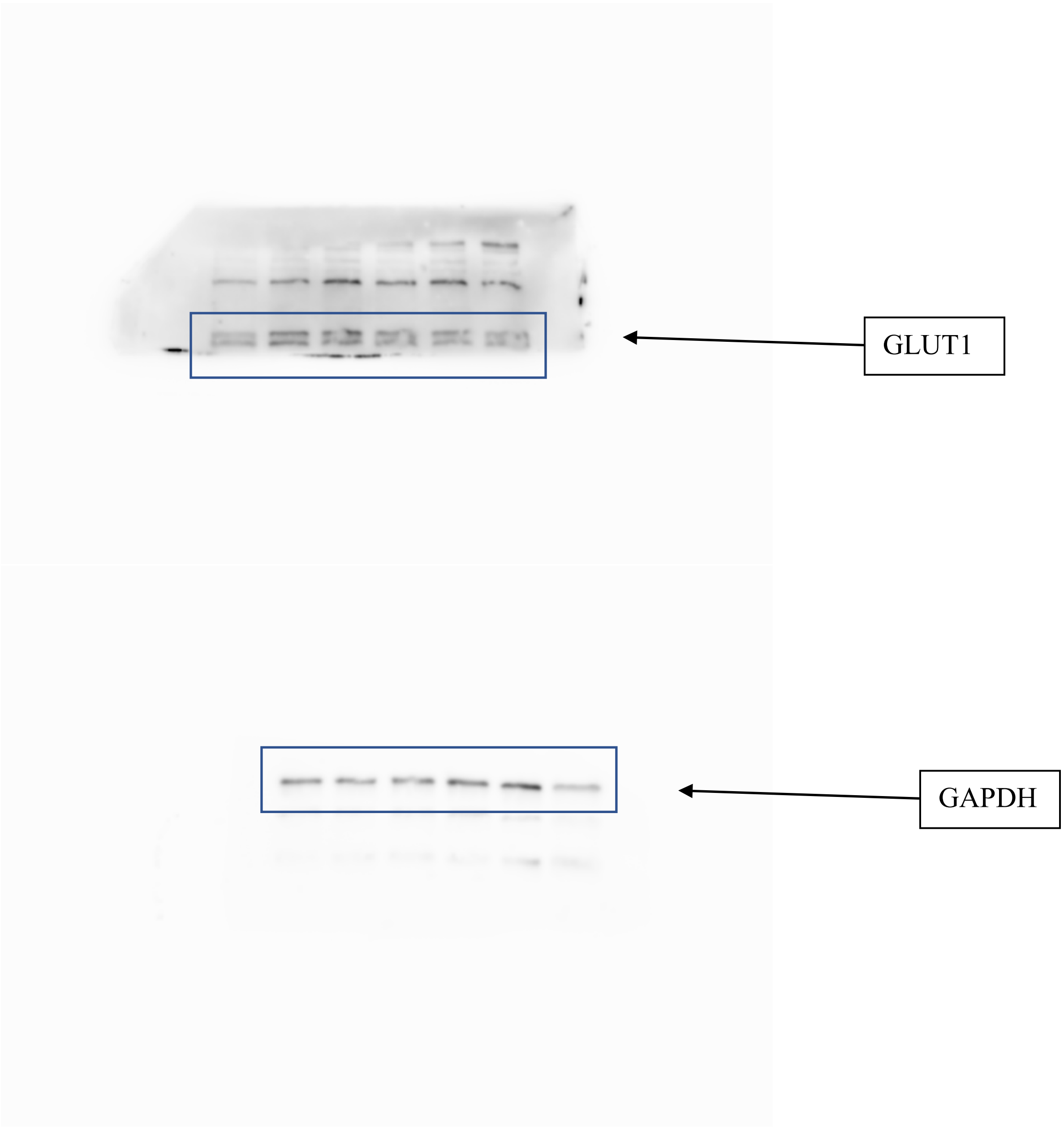


**Figure S3.** $H_2S$ modulates LPS-induced NF-κB and Akt activation and decreases LPS-induced Glut1 expression. Figure 4e was cropped from the above image.

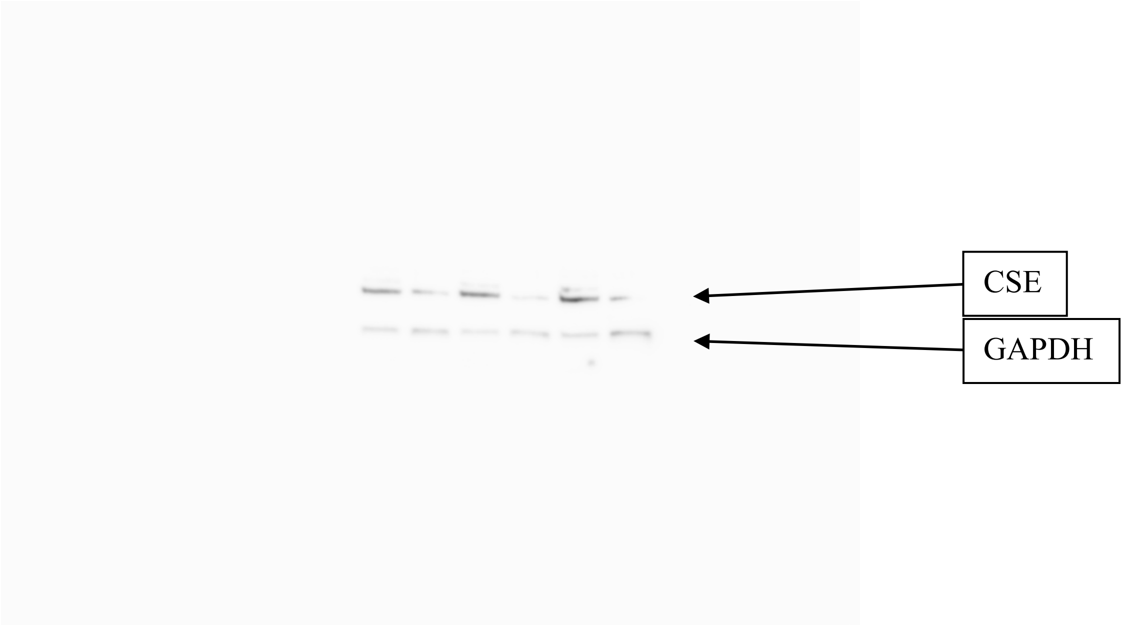


**Figure S6**. siRNA targeting CSE decreased CSE protein level in LPS-treated RAW264.7 cells. Left to right: siRNA-scramble+LPS, siRNA-CSE+LPS, siRNA-scramble+LPS, siRNA-CSE+LPS, siRNA-scramble+LPS, siRNA-CSE+LPS.
