# Supplementary material for: Cystathionine γ-lyase and hydrogen sulfide modulates glucose transporter Glut1 expression via NF-κB and PI3k/Akt in macrophages during inflammation
Source: PLoS One. 2022 Dec 15;17(12):e0278910. doi: 10.1371/journal.pone.0278910 (PMC9754168; doi:10.1371/journal.pone.0278910)
Supplement: S1 Materials — (DOCX) [file pone.0278910.s003.docx]

**Supplementary Materials:** The data presented in this study are openly available in Mendeley data at doi: 10.17632/t2mf68v374.1
